# Supplementary material for: Mapping the evolution of fertility support policies in China: A content and instrumental analysis
Source: PLoS One. 2025 Oct 9;20(10):e0332137. doi: 10.1371/journal.pone.0332137 (PMC12510515; doi:10.1371/journal.pone.0332137)
Supplement: S1 Appendix — (ZIP) [file pone.0332137.s001.zip › S1 Appendix. 226 original policy documents/40-国家卫生健康委办公厅、国家发展改革委办公厅关于做好第一批全国婴幼儿照护服务示范城市推荐申报工作的通知(FBM-CLI-4-5117602).doc]

国家卫生健康委办公厅、国家发展改革委办公厅关于做好第一批全国婴幼儿照护服务示范城市推荐申报工作的通知

**发布部门：** [国家卫生健康委员会](https://www.pkulaw.com/law/chl?Aggs.IssueDepartment=60321) [国家发展和改革委员会(含原国家发展计划委员会、原国家计划委员会)](https://www.pkulaw.com/law/chl?Aggs.IssueDepartment=60301) [机构沿革](javascript:void(0))

**发文字号：**国卫办人口函〔2022〕140号

**发布日期：**2022.04.27

**实施日期：**2022.04.27

**时效性：** [现行有效](https://www.pkulaw.com/law/chl?Aggs.TimelinessDic=01)

**效力级别：** [部门工作文件](https://www.pkulaw.com/law/chl?Aggs.EffectivenessDic=XE0303)

**法规类别：** [妇幼卫生](https://www.pkulaw.com/law/chl?Aggs.Category=09622) [老少妇幼残保护](https://www.pkulaw.com/law/chl?Aggs.Category=01604)

国家卫生健康委办公厅、国家发展改革委办公厅关于做好第一批全国婴幼儿照护服务示范城市推荐申报工作的通知
（国卫办人口函〔2022〕140号）

各省、自治区、直辖市及新疆生产建设兵团卫生健康委、发展改革委：
　　根据《国家卫生健康委 国家发展改革委[关于开展全国婴幼儿照护服务示范城市创建活动的通知](https://www.pkulaw.com/chl/3c950081cd4ea1a4bdfb.html)》（国卫人口发〔2021〕17号）要求，2022年年底前，国家卫生健康委、国家发展改革委将命名第一批全国婴幼儿照护服务示范城市（以下简称示范城市）。为做好示范城市的推荐申报工作，现将有关事项通知如下：
　　**一、**推荐数量
　　各省（区、市）要认真对照《全国婴幼儿照护服务示范城市创建标准（2022年版）》（见附件），对申报城市进行评审。申报城市必须符合10个方面的创建标准，缺一不可，特别是在多渠道发展普惠托育服务、托育机构纾困等方面出台切实管用的政策措施。向国家推荐的示范城市数量原则上不超过可参评单位总数的10%，超过10%的要排出顺序。坚持好中选优、宁缺毋滥，可以少推荐或不推荐。

　　**二、**报送材料
　　（一）城市申报报告。城市政府以正式文件的形式，向省级卫生健康委、发展改革委报送示范城市申报创建报告，对标对表2022年版创建标准，介绍主要举措、突出亮点和工作成效，简明扼要，重点突出，字数不超过3000字。
　　（二）省级评审推荐报告。省级卫生健康委、发展改革委以正式文件的形式，向国家卫生健康委、国家发展改革委报送示范城市评审推荐报告，包括本省（区、市）创建工作情况、评审推荐过程、推荐城市名单和推荐意见等，实事求是、客观真实，字数不超过2000字。
　　（三）报送时间。上述材料请于2022年9月30日前报送国家卫生健康委和国家发展改革委，电子版材料同步发至指定邮箱。报送材料前，拟推荐城市名单要在省（区、市）卫生健康委、发展改革委官方网站上公示，公示期不少于10个工作日，接受社会监督。

　　**三、**其他事项
　　（一）示范城市的创建范围、推荐申报程序和管理办法，按照国卫人口发〔2021〕17号文件要求执行。
　　（二）各省（区、市）卫生健康委、发展改革委要认真总结示范城市的经验做法，指导所辖地市对照2022年版创建标准，制定工作方案，广泛深入开展示范创建活动，不断提高婴幼儿照护服务整体水平。
　　（三）国家卫生健康委、国家发展改革委将通过实地调研、召开座谈会等方式，加强对示范城市创建活动的指导评估。
　　附件：全国婴幼儿照护服务示范城市创建标准（2022年版）

国家卫生健康委办公厅
　　国家发展改革委办公厅
　　2022年4月27日

　　附件

全国婴幼儿照护服务示范城市创建标准（2022年版）

　　**一、**建立地市级婴幼儿照护服务发展联席会议制度，将托育服务纳入民生工程或民生实事项目。

　　**二、**将发展普惠托育服务纳入城市经济社会发展“十四五”规划，制定并实施整体解决方案。

　　**三、**出台托育机构运营补贴、建设补助等具体措施，落实支持社区托育服务发展的税费优惠政策。

　　**四、**高等院校和职业院校开设婴幼儿托育服务相关专业，将托育服务人员作为急需紧缺职业（工种）纳入培训规划。

　　**五、**建立托育机构关停等特殊情况应急处置机制，针对新冠肺炎疫情等影响出台房租补贴、人员补助等纾困政策。

　　**六、**大力发展“医育结合”，在推进托育服务中加强儿童保健专业服务和指导。

　　**七、**出台并落实为家庭科学育儿提供指导服务的政策措施，提高家庭婴幼儿照护能力。

　　**八、**加大对农村婴幼儿照护服务的支持，重点关注困境儿童、留守儿童的早期发展。

　　**九、**严格落实《托育机构消防安全指南（试行）》等一系列规范性文件，近三年来没有发生涉及婴幼儿照护服务的安全责任事故。

　　**十、**2021年千人口托位数不低于全国平均水平（2.03个），且2025年千人口托位数预期值不低于4.5个。

© 北大法宝：（[www.pkulaw.com](https://www.pkulaw.com/)) 专业提供法律信息、法学知识和法律软件领域各类解决方案。北大法宝为您提供丰富的参考资料，正式引用法规条文时请与标准文本核对。
欢迎查看所有[产品和服务](https://www.pkulaw.com/helps/2.html)。法宝快讯：[如何快速找到您需要的检索结果？](https://www.pkulaw.com/helps/70.html)[法宝](https://www.pkulaw.com/helps/69.html)V6有何新特色？

扫描二维码阅读原文

**原文链接：**<https://www.pkulaw.com/chl/85ce7d53be71a1b6bdfb.html>
